# Ecological networks reveal contrasting patterns of bacterial and fungal communities in glacier-fed streams in Central Asia

**DOI:** 10.7717/peerj.7715

**Published:** 2019-09-17

**Authors:** Ze Ren, Hongkai Gao

**Affiliations:** 1 Division of Biological Sciences, University of Montana, Missoula, MT, USA; 2 Key Laboratory of Geographic Information Science (Ministry of Education), East China Normal University, Shanghai, China; 3 School of Geographic Sciences, East China Normal University, Shanghai, China

**Keywords:** Biofilm, Co-occurrence, Hydrology, Dissimilarity, Modules, Microbial community

## Abstract

Bacterial and fungal communities in biofilms are important components in driving biogeochemical processes in stream ecosystems. Previous studies have well documented the patterns of bacterial alpha diversity in stream biofilms in glacier-fed streams, where, however, beta diversity of the microbial communities has received much less attention especially considering both bacterial and fungal communities. A focus on beta diversity can provide insights into the mechanisms driving community changes associated to large environmental fluctuations and disturbances, such as in glacier-fed streams. Moreover, modularity of co-occurrence networks can reveal more ecological and evolutionary properties of microbial communities beyond taxonomic groups. Here, integrating beta diversity and co-occurrence approach, we explored the network topology and modularity of the bacterial and fungal communities with consideration of environmental variation in glacier-fed streams in Central Asia. Combining results from hydrological modeling and normalized difference of vegetation index, this study highlighted that hydrological variables and vegetation status are major variables determining the environmental heterogeneity of glacier-fed streams. Bacterial communities formed a more complex and connected network, while the fungal communities formed a more clustered network. Moreover, the strong interrelations among the taxonomic dissimilarities of bacterial community (BC) and modules suggest they had common processes in driving diversity and taxonomic compositions across the heterogeneous environment. In contrast, fungal community (FC) and modules generally showed distinct driving processes to each other. Moreover, bacterial and fungal communities also had different driving processes. Furthermore, the variation of BC and modules were strongly correlated with hydrological properties and vegetation status but not with nutrients, while FC and modules (except one module) were not associated with environmental variation. Our results suggest that bacterial and fungal communities had distinct mechanisms in structuring microbial networks, and environmental variation had strong influences on bacterial communities but not on fungal communities. The fungal communities have unique assembly mechanisms and physiological properties which might lead to their insensitive responses to environmental variations compared to bacterial communities. Overall, beyond alpha diversity in previous studies, these results add our knowledge that bacterial and fungal communities have contrasting assembly mechanisms and respond differently to environmental variation in glacier-fed streams.

## Introduction

Glaciers cover approximately 10% of the land surface on the Earth (Milner et al., 2017) and are important components of the hydrological cycle providing vital water resources (Barnett, Adam & Lettenmaier, 2005; Gardner et al., 2013; Zemp et al., 2015). However, glaciers are shrinking rapidly across the world due to accelerating global warming (Immerzeel, Van Beek & Bierkens, 2010; Sorg et al., 2012; Marzeion et al., 2014), and most of them are expected to disappear by 2050 (Zemp et al., 2006; IPCC, 2014). As a prominent component of the glacier forefront, glacier-fed streams have a highly heterogeneous environment due to longitudinal alterations of landcover, river hydrology and morphology, sediment transport, and biogeochemical processes (Hood & Scott, 2008; Laghari, 2013; Hotaling, Hood & Hamilton, 2017; Milner et al., 2017). For example, from glacier terminus to downstream, terrestrial vegetation increases (Zhang et al., 2013; Raynolds et al., 2015), stream channel lengthens (Milner, Brown & Hannah, 2009; Robinson, Thompson & Freestone, 2014), and water source compositions changes (Brown, Hannah & Milner, 2003).

Biofilms are hot spots of microbial diversity and activity in stream ecosystems (Geesey et al., 1978; Battin et al., 2016). Within stream biofilms, bacteria, fungi, and algae are the major components driving the bulk of metabolism and biogeochemical processes (Brittain & Milner, 2001; Battin et al., 2003; Von Schiller et al., 2007). The changing environment presents significant challenges for glacier-fed stream ecosystems. Previous studies have revealed that factors associated with glacier shrinkage have significant influences on the composition, diversity, and functional potential of bacterial communities in stream biofilms (Wilhelm et al., 2013, 2014; Ren, Gao & Elser, 2017; Ren et al., 2017). However, fungal communities in glacial systems are rarely studied (Edwards, 2015; Anesio et al., 2017). With the decrease in elevation, glacier coverage, and glacier source contribution to streamflow, as well as increase in distance to glacier terminus, bacterial communities showed increased alpha diversity as well as distinct taxonomic and functional compositions (Wilhelm et al., 2013, 2014; Ren, Gao & Elser, 2017; Ren et al., 2017). Biodiversity is important for generating and stabilizing ecosystem structure and functions (Loreau et al., 2001; Tilman, Isbell & Cowles, 2014). The positive effects of local species richness (alpha diversity) on ecosystem functioning have been widely confirmed by a growing number of studies (Chapin et al., 2000; Cardinale et al., 2012; Duffy, Godwin & Cardinale, 2017). However, comparing to alpha diversity, beta diversity is an underexplored facet of biodiversity (Mori, Isbell & Seidl, 2018), which accumulates from compositional variations among local assemblages and provides insights into the mechanisms underlining biodiversity changes and their ecological consequences (Anderson et al., 2011; Socolar et al., 2016). For ecological communities suffering intensive environmental fluctuations and disturbances, focusing on beta diversity is especially important (Mori, Isbell & Seidl, 2018). In addition, microorganisms in many environments often coexist in a complex network with positive and negative interactions among members, playing pivotal roles in community assembly (Fuhrman, 2009; Barberán et al., 2012; Shi et al., 2016). These interactions may imply biologically or biochemically meaningful relationships between microorganisms (Weiss et al., 2016). Microbial co-occurrence networks can reveal how taxa potentially interact with each other, how diverse taxa structure networks, and how networks are compartmentalized into modules of closely associated taxa, as well as how microbial communities responded to environmental variations (Newman, 2006; Fuhrman, 2009; De Menezes et al., 2015; Banerjee et al., 2016). In addition, modularity (the tendency of a network to contain sub-clusters of nodes) is an important ecological feature in many biological systems, providing opportunities to identify highly connected taxa and integrate high dimension data into predicted ecological modules (De Menezes et al., 2015; Shi et al., 2016). A module is defined as a group of densely connected operational taxonomic units (OTUs), which have less links with OTUs belonging to other modules (Shi et al., 2016), forming a clustered network topology (Barberán et al., 2012). Modules can help to reveal more ecological and evolutionary properties (Thompson, 2005; Olesen et al., 2007), which are easily overlooked when communities are studied as a whole or in taxonomic groups (Porter, Onnela & Mucha, 2009; Bissett et al., 2013; De Menezes et al., 2015). The relationships between microbial modules and environmental variables can improve our understanding of the influences of environmental variation on microbial community assembly (Lindström & Langenheder, 2012; De Menezes et al., 2015; Toju et al., 2016). However, previous studies in glacier-fed streams have only focused on the whole communities or certain taxonomic groups of bacteria and fungi (Robinson & Jolidon, 2005; Milner, Brown & Hannah, 2009; Wilhelm et al., 2013; Ren, Gao & Elser, 2017). The network and modularity features of bacterial and fungal communities in glacier-fed streams are remaining one of our knowledge gaps. Integrating beta diversity and network modularity can provide novel insights into assembly mechanisms of microbial communities in glacier-fed streams.

Glacier-fed streams in Tian Shan Mountains in Central Asia are particularly vulnerable to climate change, where glaciers contribute significantly to stream runoff (Aizen et al., 1997; Hagg et al., 2007; Sorg et al., 2012). Glacier shrinkage has been observed in the past decades (Farinotti et al., 2015) and will accelerate in the coming decades as temperature increases (Kraaijenbrink et al., 2017; Gao et al., 2018). Here, we investigated bacterial and fungal communities in two glacier-fed streams using high-throughput sequencing combined with hydrological modeling. We aimed to examine the microbial co-occurrence networks (considering both fungal and bacterial communities) and to assess their response patterns to environmental variation in these glacier-fed streams. In glacier foreland soil, bacterial and fungal communities had contrasting community structures and response patterns to environmental variables (Blaalid et al., 2012; Bradley, Singarayer & Anesio, 2014; Brown & Jumpponen, 2014). Glacier-fed streams have intimate connections with terrestrial ecosystems in the glacier foreland through multiple ways (Crump, Amaral-Zettler & Kling, 2012; Gutiérrez et al., 2015; Ruiz-González, Niño-García & Del Giorgio, 2015). Thus, we hypothesize that bacterial and fungal communities in glacier-fed streams have contrasting assembly mechanisms and respond differently to environmental variation.

## Materials and Methods

### Study area

**Figure 1:**
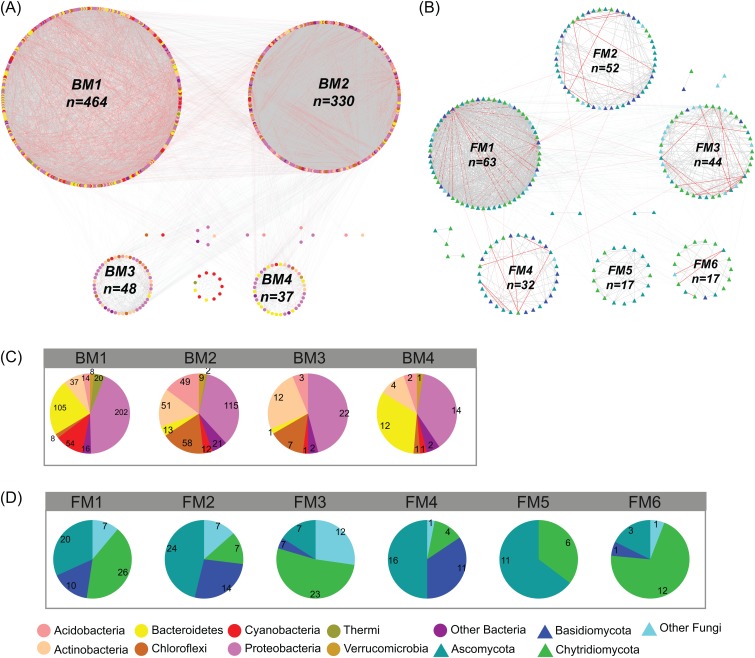
Map of the study area. (A) Location of the study area. (B) Elevation distribution of the study area. GN1 represents Urumqi Glacier No. 1. (C) The Normalized difference of vegetation index (NDVI) of the study area.

The Tian Shan mountains, known as the “water tower of Central Asia,” span a large area of Central Asia from northwestern China to southeastern Kazakhstan and from Kyrgyzstan to Uzbekistan (Fig. 1). In Tian Shan area, glaciers contribute considerably to water resources and play an important role in streamflow regimes (Sorg et al., 2012; Unger-Shayesteh et al., 2013). In China’s portion of the Tian Shan Mountains, there are 7,934 glaciers with a total area of 7,179 km^2^ and a total volume of 707 km^3^ (Guo et al., 2014). However, glaciers in the Tian Shan Mountains are extremely sensitive to global warming (Kraaijenbrink et al., 2017) and have been shrinking rapidly due to climate warming since the 1970s (Narama et al., 2010; Farinotti et al., 2015). For example, Urumqi Glacier No. 1 (GN1, 43°06′N, 86°49′E) is located in eastern Tian Shan Mountain at the headwater of the Urumqi River (Fig. 1) and retreated from an area of 1.95 km^2^ in 1962 to 1.65 km^2^ in 2010 (Zhang et al., 2014). In 2050, GN1 will likely lose up to 54% of the glacier area and 79% of the ice volume relative to 1980 (Gao et al., 2018).

### Field sampling

In June 2016, we investigated two glacier-fed streams in the Tian Shan Mountain. Water samples and benthic biofilm samples were collected from 11 sample sites in total spanning from the elevation of 3,828 to 2,646 m (Fig. 1). The sample sites were chosen along the streams in order to have heterogeneous environments, including different land vegetation and hydrological properties such as glacier contributions to stream flow. However, due to the constraint of accessibility, the sites were not spaced at equal intervals. At each sample site, six to nine submerged rocks were randomly sampled from the stream cross section below 10 cm. A sterilized nylon brush was used to remove the benthic biofilm from each stone in an area of 4.5 cm diameter on the upper surface. The slurry was rinsed with 500 mL sterile water. Approximately 10 mL of the mixed slurry was filtered through a 0.2-μm polycarbonate membrane filter (Whatman, Maidstone, UK) which was immediately frozen in liquid nitrogen in the field. After transported to the lab, the benthic biofilm samples were stored under −80 °C until DNA extraction. In addition, 500 mL water samples were collected for chemical analyses with three replications and stored under 4 °C.

### Environmental factors

At each sample site, pH, conductivity (Cond), and elevation were measured in situ using a handheld pH meter (PHI 400 Series; Beckman Coulter, Brea, CA, USA), YSI meter (model 80, Yellow Springs, OH, USA), and GPS unit (Triton 500; Magellan, Santa Clara, CA, USA), respectively. Unfiltered water samples were directly used to measure total nitrogen (TN) and total phosphorus (TP). TN was analyzed by ion chromatography with prior persulfate oxidation (EPA 300.0). TP was analyzed using the ammonium molybdate method with prior oxidation (EPA 365.3). Filtered water samples (filtered with pre-combusted GF/F filters) were used to test nitrate (NO_3_^−^), ammonium (NH_4_^+^), soluble reactive phosphorus (SRP), and dissolved organic carbon (DOC). NO_3_^−^ was analyzed using ion chromatography (EPA 300.0). NH_4_^+^ was analyzed using the indophenol colorimetric method (EPA 350.1). SRP was measured according to the ammonium molybdate method (EPA 365.3). DOC was measured using a total organic carbon (TOC) analyzer (TOC-VCPH; Shimadzu Scientific Instruments, Columbia, MD, USA). Water chemistry data was reported in our previous research (Ren, Gao & Elser, 2017).

In glacier-fed streams, both biotic and abiotic environments are tightly linked to the relative contributions of glacier melt and runoff to the stream flow (Milner et al., 2001; Hannah et al., 2007; Kuhn et al., 2011). According to the landscape-based hydrological model proposed by Gao et al. (2016, 2017), we classified the landscape into glaciated and non-glaciated. For each sample site, the proportion of glaciated area (GA) in its sub-catchment was calculated and the proportion of glacier source water (GS) in the total runoff was derived from the model (Gao et al., 2016, 2017). The hydrological distance to glacier terminus (GD) was measured according to the river channel network (Fig. 1). These hydrological parameters were also reported in our previous research (Ren, Gao & Elser, 2017).

In the study area, the vegetation (grassland) status was measured by the normalized difference vegetation index (NDVI) using the Terra moderate resolution imaging spectroradiometer Vegetation Indices (MOD13Q1) Version 6 data downloaded from USGS (https://earthexplorer.usgs.gov/) (Fig. 1). The MOD13Q1 product was generated on June 26, 2017 and has a resolution of 250 m. NDVI is calculated based on the absorption of red light and the reflection of infrared radiation by vegetation (Rouse et al., 1974). The equation is represented as NDVI = (NIR − RED)/(NIR + RED), where NIR is near infrared reflectance and RED is visible red reflectance. It has been demonstrated that NDVI exhibits close relationships with above-ground vegetation biomass and coverage (Carlson & Ripley, 1997; Eastwood et al., 1997; Ren et al., 2019). For each stream site, the average NDVI of its sub-catchment (the upstream area of the stream site) was calculated as the mean NDVI of each pixel in the sub-catchment.

### DNA extraction, PCR, and sequencing

Bacterial 16S rRNA gene sequences and fungal 18S rRNA gene sequences were analyzed to determine the bacterial community (BC) and fungal community (FC), respectively. To determine the fungal community, the internal transcribed spacer regions and 18S rRNA genes are commonly used and provide similar results and congruent conclusions (Brown, Rigdon-Huss & Jumpponen, 2014). We used 18S rRNA gene sequencing to detect the fungal community in this study. DNA was extracted using the PowerSoil DNA Isolation Kit (MoBio, Carlsbad, CA, USA) following the manufacturer’s protocol. The V3–V4 regions of the 16S rRNA genes were amplified using the bacterium-specific forward and reverse primers 338F-ACTCCTACGGGAGGCAGCA and 806R-GGACTACHVGGGTWTCTAAT (Invitrogen, Vienna, Austria) (Huws et al., 2007; Masoud et al., 2011; Caporaso et al., 2012). The V4–V5 regions of the 18S rRNA genes were amplified using the fungus-specific forward and reverse primers 817F-TTAGCATGGAATAATRRAATAGGA and 1196R-TCTGGACCTGGTGAGTTTCC-3′ (Invitrogen, Vienna, Austria) (Borneman & Hartin, 2000). The forward primers were barcoded, and the barcodes were designed considering the balanced guanine–cytosine content, minimal homopolymer runs, and no self-complementarity of more than two bases to reduce internal hairpin propensity (Hawkins et al., 2018). PCR reaction systems were prepared using a Premix Taq Kit (Code No. RR902A; Takara, Kusatsu,, Japan) according to the manufacturer’s instructions. The total volume of each PCR reaction was 20 μL, containing 10 μL of 2×EX Premix Taq™ Polymerase, one μL of forward primer, one μL of reverse primer, one μL of NDA extraction, and seven μL of Nuclease-free water. The PCR reactions were conducted with a thermal cycler (ABI 2700, SeqGen, Torrance, CA, USA) with a temperature profile of 1-min hot start at 80 °C, followed by pre-denaturation at 94 °C for 5 min, 30 cycles of amplification (denaturation at 94 °C for 30 s, annealing at 52 °C for 30 s, and extension at 72 °C for 90 s), and a final extension at 72 °C for 10 min. The PCR amplicons were verified in 1.0% agarose with 1× TAE buffer using electrophoresis, purified using the Gel Extraction Kit (Qiagen, Hilden, Germany), and quantified by Qubit 2.0 Fluorometer (Invitrogen, Carlsbad, CA, USA). One of the fungi sample (N2) was not successfully amplified. The purified and quantified DNA libraries were then pooled together according to their concentrations. The pooled library was sequenced on an Illumina MiSeq (PE300) sequencing platform.

### Analyses

Raw sequence data of bacterial 16S rRNA (available at NCBI, PRJNA398147, SRP115356) and fungal 18S rRNA (available at NCBI, PRJNA542974, SRP198430) were processed using QIIME 1.9.0 (Caporaso et al., 2010). The forward and reverse reads were merged. The merged sequences were then assigned to samples based on the barcode. The barcode and primer sequence were cut off to truncate the sequences. The sequences with length >200 bp and mean quality score <20 were discarded. Using UCHIME algorithm (Edgar et al., 2011), the chimeric sequences were detected and removed. Finally, the effective sequences of 16S and 18S rRNA were grouped into OTUs against the Silva 132 database at 99% threshold.

All the analyzed environmental variables, including GD, GA, GS, NDVI, elevation, pH, Cond, TN, NO_3_^−^, NH_4_^+^, TP, SRP, and DOC, were standardized using the “normalize” method in *decostand* function in vegan 2.5-3 package (Oksanen et al., 2007). Spearman correlation analyses (*cor* function in stats v3.6.0 package) were used to assess the pairwise relationships between environmental variables and visualized using *corrplot* function in corrplot 0.84 package. Mantel tests (*mantel* function in vegan 2.5-3 package) were used to assess the relationships among spatial dissimilarity (represented by geographic distance), overall environmental distance, hydrological dissimilarity, nutrient dissimilarity, and vegetation dissimilarity. Partial Mantel tests (*mantel.partial* function in vegan 2.5-3 package) were used to assess those relationships by controlling nutrient dissimilarity. The geographic distance was calculated based on the GPS coordinates of sample sites using *distGeo* function in geosphere 1.5-10 package. The environmental distance was represented by Euclidean distance based on all analyzed environmental variables. Hydrological dissimilarity was represented by Euclidean distance based on GD, GA, and GS. Nutrient dissimilarity was represented by Euclidean distance based on TN, NO_3_^−^, NH_4_^+^, TP, and SRP. Vegetation dissimilarity was represented by Euclidean distance based on NDVI. Euclidean distances were calculated using *vegdist* function in vegan 2.5-3 package (Oksanen et al., 2007). All the above statistical analyses were conducted in R 3.5.1 (R Development Core Team, 2017).

The co-occurrence networks of bacterial and fungal communities were assessed and visualized using Cytoscape (version 3.7.1) (Shannon et al., 2003). In network analyses, the pairwise correlations between OTUs (OTUs with a relative abundance >0.01%) were calculated using Spearman’s correlation based on the relative abundance of OTUs (data was transformed by Hellinger transformation using *decostand* function in vegan 2.5-3 package). Only strong (*R* > 0.7 OR *R* < −0.7) and significant (*P* < 0.01) correlations were considered in network analysis. ClusterMaker app (Morris et al., 2011) was used to analyze the modular structures of the co-occurrence networks. Modularity values greater than 0.4 suggest that the network has a modular structure (Newman, 2006). The group attributes layout algorithm was used to construct the networks based on modules. The basic topological metrics of networks were calculated, including number of nodes, number of edges, clustering coefficient, characteristic path length, network density, network heterogeneity, and modularity. The taxonomic dissimilarities (beta diversity) of the BC and FC were calculated based on Bray–Curtis distance in terms of the relative abundance of OTUs using R package vegan 2.5-3 (Oksanen et al., 2007). Moreover, the taxonomic dissimilarities of the major modules (modules have more than 15 nodes) were also calculated based on Bray–Curtis distance in terms of the relative abundance of OTUs in the module. BM1, BM2, BM3, and BM4 represent four major modules in the bacterial network. FM1, FM2, FM3, FM4, FM5, and FM6 represent six major modules in the fungal network. Mantel tests were used to assess the correlations between spatial and environmental dissimilarities and taxonomic dissimilarities of bacterial and fungal communities and modules. The relationships among different modules and communities for bacteria and fungi were also assessed using Mantel tests.

## Results

### Environmental variations and community taxonomic dissimilarities

**Figure 2:**
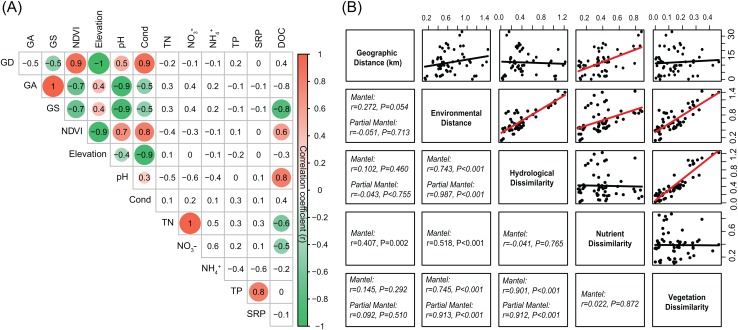
(A) Pairwise-Spearman correlations between environmental variables. The magnitude of correlation coefficient is only shown graphically with color scale when correlation is significant (*P* < 0.05). The color intensity and the size of the circle are proportional to the correlation coefficients. (B) Correlations between the different components of environmental dissimilarities. Spearman correlations (*r*) and associated *P*-values were calculated by Mantel test. Nutrient dissimilarity was controlled in Partial Mantel test.

**Figure 3:**
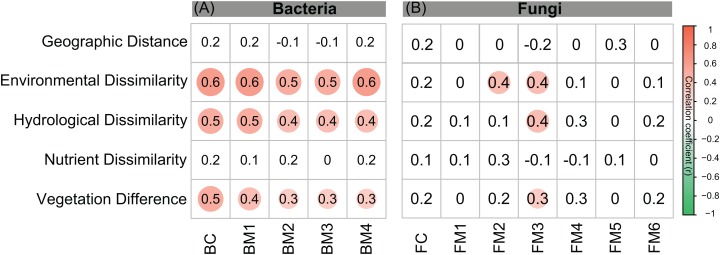
Correlations between different components of environmental distance (environmental dissimilarity, hydrological dissimilarity, nutrient dissimilarity, and vegetation dissimilarity) and taxonomic dissimilarities of (A) bacterial and (B) fungal communities and modules. Spearman correlations were calculated by Mantel test. The magnitude of correlation coefficient is only shown graphically with color scale when correlation is significant (*P* < 0.05). The color intensity and the size of the circle are proportional to the correlation coefficients.

The environmental variables of the streams varied across the sampling sites and showed differing interrelationships (Fig. 2). GD, GA, GS, NDVI, elevation, pH, and conductivity were closely correlated with each other (Fig. 2A). DOC was negatively correlated with GS, TN, and NO_3_^−^, while positively correlated with NDVI and pH (Fig. 2A). Across the sampling sites, hydrological and vegetation dissimilarities had strong linear relationships with overall environmental dissimilarity, while nutrient dissimilarity only had a weak relationship (Fig. 2B). Moreover, hydrological and vegetation dissimilarities had a strong interrelationship with each other but without significant relationships to nutrient dissimilarity (Fig. 2B). Geographic distance only had a significant relationship with nutrient dissimilarity (Fig. 2B). In addition, Mantel tests showed that environmental, hydrological, and vegetation dissimilarities had significant positive relationships to taxonomic dissimilarities of BC but not to FC (Fig. 3). However, geographic distance and nutrient dissimilarity did not have significant relationships with taxonomic dissimilarities of both bacterial and fungal communities (Fig. 3).

### Bacteria and fungi co-occurrence patterns and modular structures

**Figure 4:**
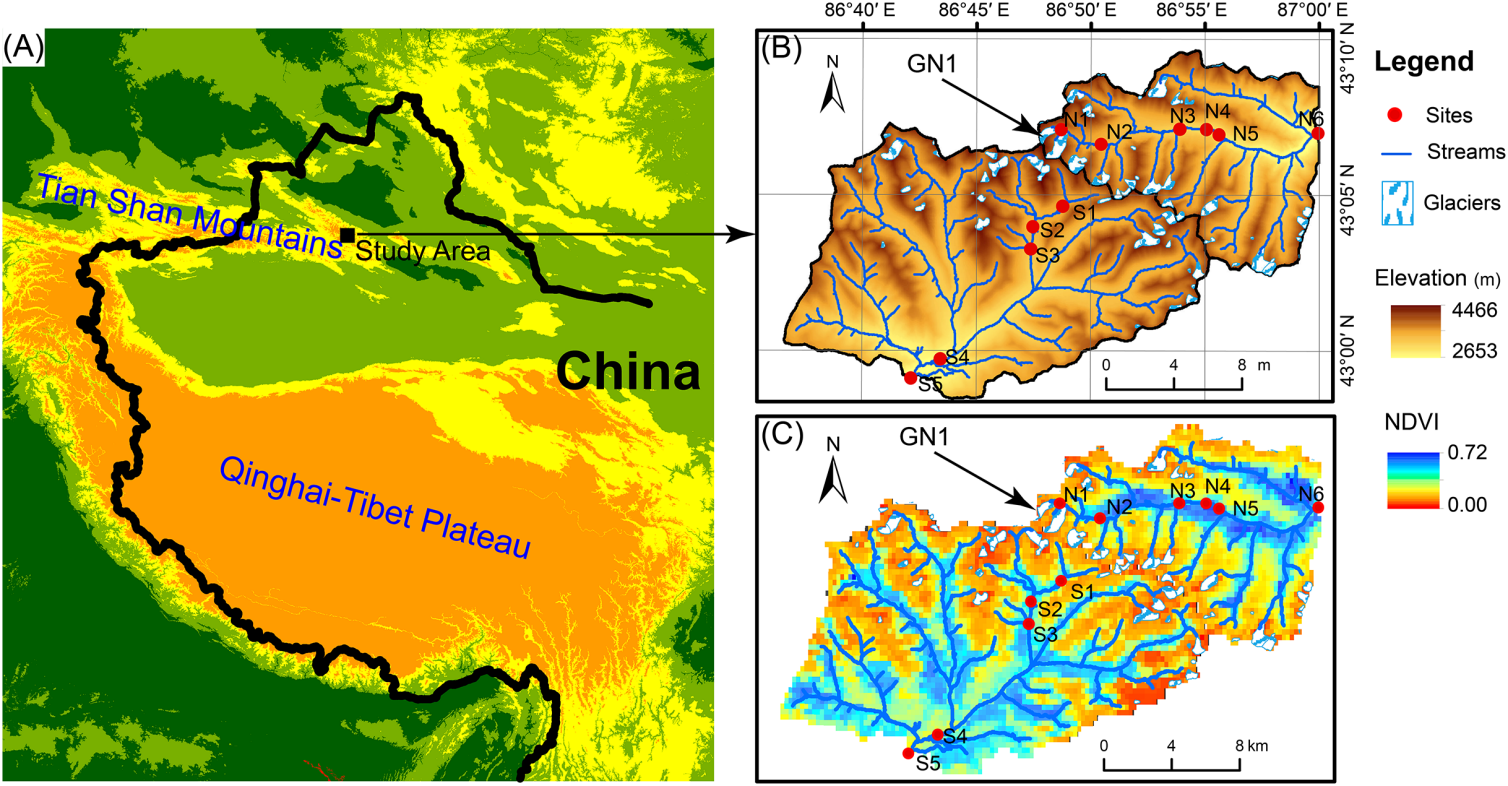
Co-occurrence network of (A) bacterial and (B) fungal communities. Dot and triangle represent bacterial and fungal OTUs (OTUs with a relative abundance >0.01%), respectively. OTUs were colored according to major phylum (phylum with relative abundance >1%). Edges represent Spearman correlation relationships (*P* < 0.01). Gray lines indicate positive associations and pink lines indicate negative associations. The circles represent modules of the networks. The pie graphs in the below two panels show the composition of the modules in (C) bacterial and (D) fungal networks with the number representing the number of OTUs.

In the studied glacier-fed streams, bacteria and fungi formed complex co-occurrence networks (Fig. 4). The bacterial and fungal networks consisted of 904 and 238 nodes and 21,463 and 1,348 edges, respectively (Table 1). The bacterial network had a higher number of nodes and edges as well as a higher network density (Fig. 4; Table 1), indicating the bacterial network was more complex and connected than the fungal network. However, the fungal network exhibited a higher clustering coefficient, characteristic path length, network heterogeneity, and modularity, indicating that the fungal network had a more clustered topology than the bacterial network (Fig. 4; Table 1).

**Table 1:** Topological parameters of the network of bacterial and fungal communities.

Topological parameters	Description	Bacterial	Fungal
Number of nodes	Number of OTUs in the network	904	238
Number of edges (in total)	Strong and significant correlations	21,463	1,348
Number of edges (positive)	Positive correlations	18,419	1,253
Number of edges (negative)	Negative correlations	3,044	95
Clustering coefficient	The fraction of observed vs. possible clusters for each node	0.440	0.451
Characteristic path length	The median of the means of the shortest path lengths connecting each vertex to all other vertices	2.887	3.829
Network density	The ratio of the number of edges and the number of possible edges	0.053	0.048
Network heterogeneity	Density distribution of connections between nodes	0.929	1.107
Modularity	Tendency of a network to contain sub-clusters of nodes	0.52	0.599

**Figure 5:**
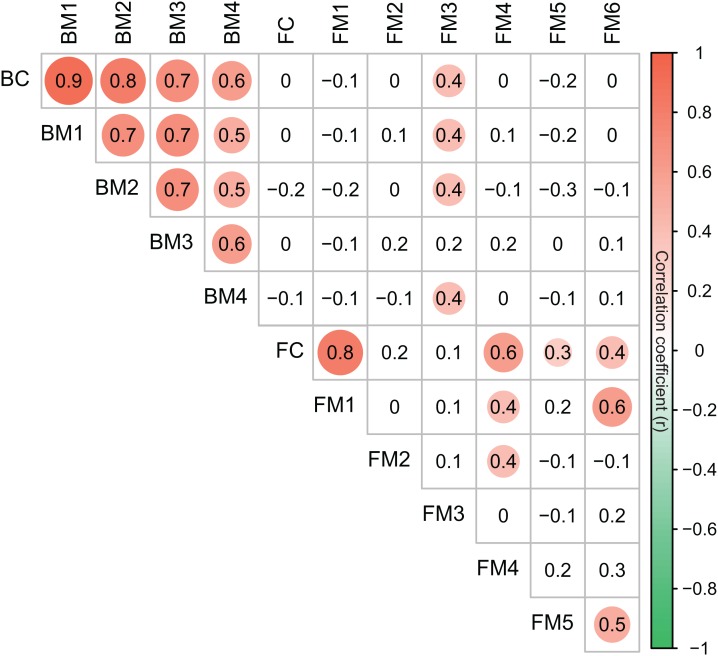
Correlation matrix of the taxonomic dissimilarities for bacterial and fungal communities and modules. The magnitude of correlation coefficient is only shown graphically with color scale when correlation is significant (*P* < 0.05). The color intensity and the size of the circle are proportional to Spearman correlation coefficients.

Co-occurrence networks can be compartmentalized into modules within which nodes are closely associated and are expected to share environmental preferences. We found that the OTUs in bacterial and fungal networks were grouped into four and six major modules (modules with more than 15 nodes), respectively (Figs. 4A and 4B). All the modules were formed by various microbial taxa (Figs. 4C and 4D), which shown differently across sample sites (Figs. S1 and S2). Modules had significantly different taxonomic compositions to each other (Tables S1 and S2). Mantel tests showed that the taxonomic dissimilarities of the BC and modules had strong interrelationships (Fig. 5). The taxonomic dissimilarities of FC and modules only had weak interrelationships. Between bacterial and fungal networks, only FM3 had strong relationships with BC and BMs. Mantel tests also revealed that all bacterial modules (BM1, BM2, BM3, and BM4) and one fungal module (FM3) were positively correlated with environmental, hydrological, and vegetation dissimilarities, but not with geographic distance and nutrient dissimilarity (Fig. 3).

## Discussion

Bacterial and fungal communities in stream biofilms are major components of glacier-fed stream ecosystems. The strong correlations between bacterial communities and environmental, hydrological, and vegetation characteristics suggest substantial influences of spatial heterogeneity and potential influences of glacier shrinkage on bacterial communities (Fig. 3). However, the variation of bacterial communities was not associated with stream nutrient variations and geographic distance. In our studied glacier-fed streams, the proportion of GA, the proportion of glacier source water (GS), the hydrological distance to glacier terminus (GD)and the NDVI were the environmental variables associated with longitudinal patterns of glacier-fed streams. Glacier-fed streams are fed by various sources, including ice-melt, snowmelt, and groundwater (Brown, Hannah & Milner, 2003). The contribution of different water sources varies longitudinally from the glacier terminus to downstream reaches and temporally with glacier shrinkage (Brittain & Milner, 2001; Milner, Brown & Hannah, 2009; Gao et al., 2016), resulting in distinct hydrology and physicochemical features which control the ecological structures and processes in glacier-fed streams (Brown, Hannah & Milner, 2003, 2007; Sertic Peric et al., 2015). Synchronizing with the hydrological changes in glacier-fed streams, vegetation growth and aboveground biomass in the catchment also has a clear elevation gradient (Carlyle, Fraser & Turkington, 2014) which will likely be amplified due to climate warming (Zhang et al., 2013; Raynolds et al., 2015). The changed landcover modifies terrestrial and aquatic biogeochemistry (Sadro, Nelson & Melack, 2012), and affects stream biofilms (Figueiredo et al., 2010; Nielsen et al., 2012). Thus, hydrological variables (GD, GA, and GS) and vegetation variable (NDVI) determine the environmental heterogeneity of glacier-fed streams and can potentially indicate glacier shrinkage. It has been widely demonstrated that glacier shrinkage alters watershed landcover and instream environments (Hood & Scott, 2008; Jacobsen & Dangles, 2012; Laghari, 2013; Raynolds et al., 2015; Milner et al., 2017), imposing impacts on bacterial communities (Nelson, Sadro & Melack, 2009; Hotaling, Hood & Hamilton, 2017). For example, the alpha diversity of biofilm bacteria decreased with the increases of elevation, the proportion of glacier area in the watershed, and the relative contribution of glacier sources to stream runoff (Wilhelm et al., 2013; Ren, Gao & Elser, 2017). Potential functions of bacterial communities are also significantly associated with hydrological factors (Ren et al., 2017). Our results further suggest strong influences of hydrological and vegetation characteristics on bacterial communities, leading to more different (biotic heterogenization) bacterial communities in glacier-fed streams.

In contrast to bacterial communities, environmental, hydrological, and vegetation dissimilarities were not significantly associated with the dissimilarity of fungal communities. Moreover, the variation fungal communities were also not associated to nutrient variations. The results suggest that FC variations were not affected by the environmental variation and might be insensitive to glacier shrinkage. We proposed that the unique responses of fungi may relate to the low temperature which can suppress the response of fungal communities to environmental variation. Fungi can survive and grow in harsh conditions with low temperatures such as glacier and snow by evolving various adaptive features (Hassan et al., 2016). These fungi are known as psychrophiles and psychrotrophs. Although they exist widely in cold environments, the optimum temperature for the growth of psychrophilic fungi is around 15 °C and for psychrotrophic fungi is 20 °C (Gounot, 1986; Robinson, 2001; Cavicchioli et al., 2002; Turchetti et al., 2008). In glacier-fed streams, water temperature is usually below 10 °C or even close to 0 °C in summer (Milner & Petts, 1994). More interestingly, the contrasting response patterns of bacterial and fungal communities were also found in glacier foreland soil ecosystems, where bacterial communities are strongly influenced by the presence of vegetation and environmental heterogeneity and show convergence (Brown & Jumpponen, 2014). In contrast to the bacterial communities in glacier foreland soil, fungal richness and diversity were more static and the community structure and distribution show a large extent of stochastic processes across the glacier foreland (Blaalid et al., 2012; Bradley, Singarayer & Anesio, 2014; Brown & Jumpponen, 2014). It has been revealed that bacteria and fungi in headwater streams are similar to communities in adjacent soil due to intimate associations between headwater streams and terrestrial ecosystems (Crump, Amaral-Zettler & Kling, 2012; Gutiérrez et al., 2015; Ruiz-González, Niño-García & Del Giorgio, 2015). The immigration and advection of allochthonous bacteria and fungi from terrestrial environments can influence bacterial and fungal communities in glacier-fed streams (Crump, Amaral-Zettler & Kling, 2012; Gutiérrez et al., 2015). The unique response of fungal communities in glacier-fed streams is congruent with the observations in the periglacial soils, suggesting differing trajectories of fungal and BC variations in glacier-fed streams.

The different patterns of bacterial and fungal communities in glacier-fed streams were further supported by network analysis, which showed that the bacterial communities formed a more complex and connected network, while the fungal communities formed a more clustered network (Fig. 4; Table 1). Microbial communities are complex assemblages comprised by highly interactive taxa (Fuhrman, 2009; Jones, Hambright & Caron, 2018). This study is the first to explore the organization of the bacterial and fungal communities in glacier-fed streams using a co-occurrence approach with consideration of the driving forces. In general, communities with tight co-occurrence interactions and high complexity have a lower stability and are more susceptible to disturbance (Montoya, Pimm & Sole, 2006; Saavedra et al., 2011). The highly connected and complex bacterial network suggests that bacterial communities in the glacier-fed streams were more sensitive to environmental variations, especially to instream hydrological properties and land vegetation. Moreover, in a complex network, the highly interconnected species are grouped into a module (Barabási & Oltvai, 2004; Newman, 2006). The strong interrelations among the taxonomic dissimilarities of BC and modules suggest that they had common processes in driving diversity and composition across various environments (Delgado-Baquerizo et al., 2018). In contrast, FC and modules generally had distinct driving processes to each other. Moreover, the fungal and bacterial communities also had different driving processes (except FM3 and bacterial communities and modules). Consistent with these findings, the variation of all bacterial modules and FM3 were strongly associated with environmental variation except nutrient dissimilarity, while fungal modules (except FM3) did not respond to environmental variation. Thus, our results suggest that, in our studied area, environmental variation had strong influences on bacterial communities and their assembly mechanisms but not on fungal communities (except one module) in biofilms of glacier-fed streams. The fungal communities may have unique assembly mechanisms which lead to their insensitive responses to environmental variations.

## Conclusion

Glacier shrinkage imposes significant influences on glacier-fed streams. Integrating beta diversity, our study provides the first co-occurrence network analyses of bacterial and fungal communities in glacier-fed streams. Firstly, this study highlighted that hydrological variables and vegetation status are important components in determining environment heterogeneity of glacier-fed streams and are indicator variables of glacier shrinkage. Then we identified co-occurrence properties of the microbial communities and their responses to environmental variations. Bacterial communities formed a more complex and connected network, while the fungal communities formed a more clustered network. Nutrients were insignificant to the assemblies of both bacterial and fungal communities in these glacier-fed streams. However, hydrological properties and vegetation status impose significant influences on assemblies of the BC but not on the FC. The results suggest the influences of glacier shrinkage on bacterial communities. However, fungi communities might be insensitive to glacier shrinkage. The results would add our knowledge of microbial community assembly mechanisms and the responses of microbial communities to environmental variations caused by glacier shrinkage.

## Supplemental Information

10.7717/peerj.7715/supp-1 Supplemental Information 1 Supplementary Figures. Figure S1 Distributions of OTUs across sample sites for bacterial modules.
Figure S2 Distributions of OTUs across sample sites for fungal modules.

10.7717/peerj.7715/supp-2 Supplemental Information 2 Supplementary Tables. Table S1 Pairwise dissimilarity tests of taxonomic composition among bacterial modules using PERMANOVA (*adonis* function in vegan package 2.5-3). *R*^2^ and *P*-values (in bracket) are shown.
Table S2 Pairwise dissimilarity tests of taxonomic composition among fungal modules using PERMANOVA (*adonis* function in vegan package 2.5-3). *R*^2^ and *P*-values (in bracket) are shown.

## Additional Information and Declarations

### Data Availability

The following information was supplied regarding data availability: Data is available at NCBI: SRP115356 and SRP198430.
