# Supplementary material for: Ecological networks reveal contrasting patterns of bacterial and fungal communities in glacier-fed streams in Central Asia
Source: PeerJ. 2019 Sep 17;7:e7715. doi: 10.7717/peerj.7715 (PMC6753927; doi:10.7717/peerj.7715)

## Supplementary Figures

Figure S1 Distributions of OTUs across sample sites for bacterial modules


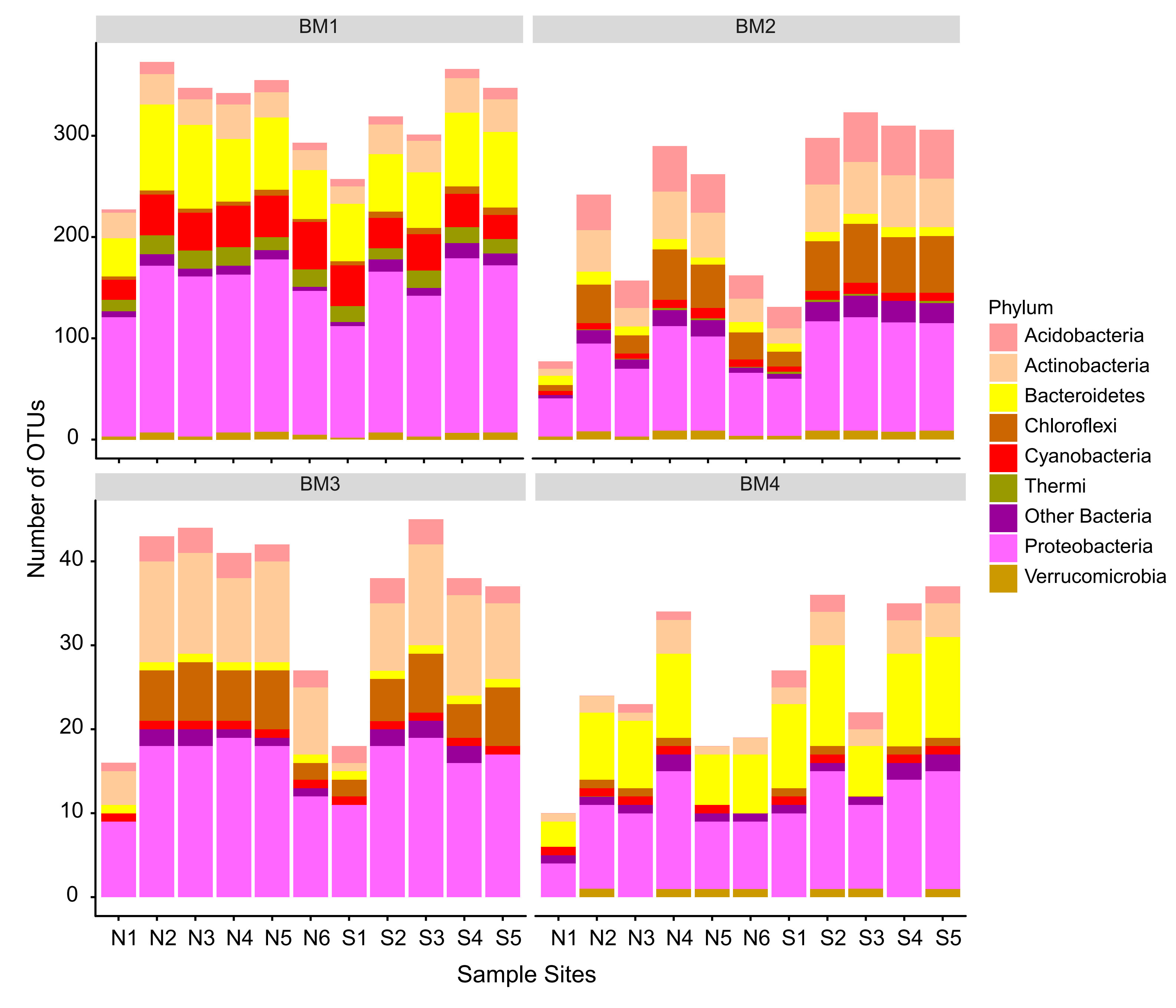


Figure S2 Distributions of OTUs across sample sites for fungal modules


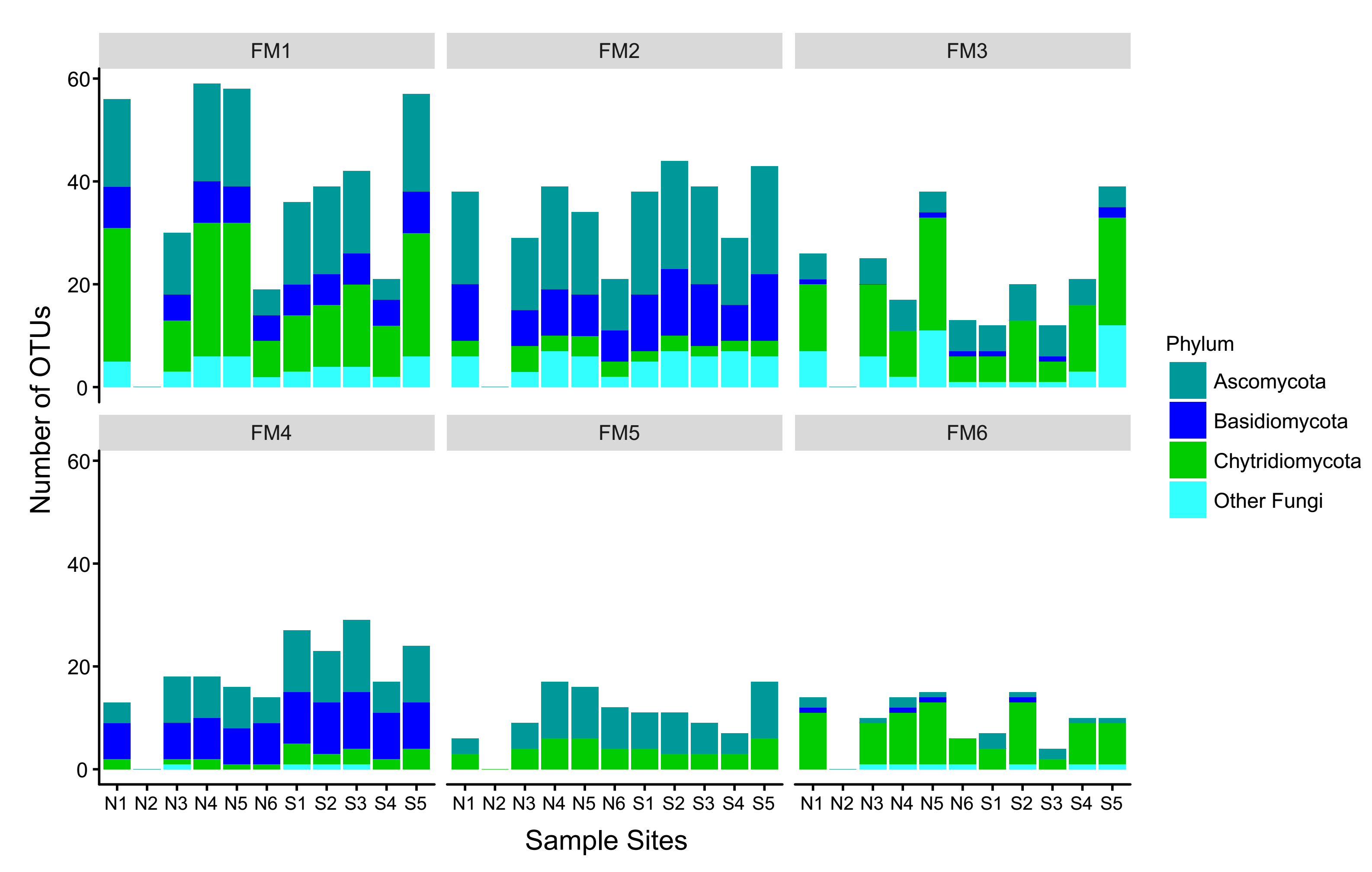

Supplement: Supplemental Information 1 — Figure S1 Distributions of OTUs across sample sites for bacterial modules. Figure S2 Distributions of OTUs across sample sites for fungal modules. [file peerj-07-7715-s001.docx]
