# Supplementary material for: Ecological networks reveal contrasting patterns of bacterial and fungal communities in glacier-fed streams in Central Asia
Source: PeerJ. 2019 Sep 17;7:e7715. doi: 10.7717/peerj.7715 (PMC6753927; doi:10.7717/peerj.7715)
Supplement: Supplemental Information 2 — Table S1 Pairwise dissimilarity tests of taxonomic composition among bacterial modules using PERMANOVA (adonis function in vegan package 2.5-3). R2 and P-values (in bracket) are shown. Table S2 Pairwise dissimilarity tests of taxonomic composition among fungal modules using PERMANOVA (adonis function in vegan package 2.5-3). R2 and P-values (in bracket) are shown. [file peerj-07-7715-s002.docx]

## Supplementary Tables

Table S1 Pairwise dissimilarity tests of taxonomic composition among bacterial modules using PERMANOVA (*adonis* function in vegan package 2.5-3). R^2^ and P-values (in bracket) are shown.

|  | BM1 | BM2 | BM3 |
| --- | --- | --- | --- |
| BM2 | 0.553  (<0.001) |  |  |
| BM3 | 0.553  (<0.001) | 0.563  (<0.001) |  |
| BM4 | 0.559  (<0.001) | 0.568  (<0.001) | 0.561  (<0.001) |

Table S2 Pairwise dissimilarity tests of taxonomic composition among fungal modules using PERMANOVA (*adonis* function in vegan package 2.5-3). R^2^ and P-values (in bracket) are shown.

|  | FM1 | FM2 | FM3 | FM4 | FM5 |
| --- | --- | --- | --- | --- | --- |
| FM2 | 0.358  (<0.001) |  |  |  |  |
| FM3 | 0.441  (<0.001) | 0.516  (<0.001) |  |  |  |
| FM4 | 0.374  (<0.001) | 0.442  (<0.001) | 0.537  (<0.001) |  |  |
| FM5 | 0.273  (<0.001) | 0.331  (<0.001) | 0.411  (<0.001) | 0.347  (<0.001) |  |
| FM6 | 0.345  (<0.001) | 0.410  (<0.001) | 0.501  (<0.001) | 0.428  (<0.001) | 0.319  (<0.001) |
